# Supplementary material for: EphrinB2-EphB4 signalling provides Rho-mediated homeostatic control of lymphatic endothelial cell junction integrity
Source: eLife. 2020 Sep 8;9:e57732. doi: 10.7554/eLife.57732 (PMC7478896; doi:10.7554/eLife.57732)
Supplement: Transparent reporting form [file elife-57732-transrepform.pdf]

eLIFE
1st Floor
24 Hills Road
Cambridge CB2 1JP, UK
P 01223 855340
W elifesciences.org
T @elife

eLife's transparent reporting form

We encourage authors to provide detailed information *within their submission* to facilitate the interpretation and replication of experiments. Authors can upload supporting documentation to indicate the use of appropriate reporting guidelines for health-related research (see EQUATOR Network), life science research (see the BioSharing Information Resource), or the ARRIVE guidelines for reporting work involving animal research. Where applicable, authors should refer to any relevant reporting standards documents in this form.

If you have any questions, please consult our Journal Policies and/or contact us: editorial@elifesciences.org.

Sample-size estimation

- You should state whether an appropriate sample size was computed when the study was being designed
- You should state the statistical method of sample size computation and any required assumptions
- If no explicit power analysis was used, you should describe how you decided what sample (replicate) size (number) to use

Please outline where this information can be found within the submission (e.g., sections or figure legends), or explain why this information doesn't apply to your submission:

We have not computed the sample size but it was determined based on our previous experience, to allow statistical analysis of data, and in accordance with similar previously published studies. All *in vivo* functional data and quantifications are based on a minimum of 3 mice per condition, except for quantifications in Fig 2C, D, Fig 2-supplement figure 3 and 3F, which were analysed for 2 mice per condition (with n=250 junctions, n=150 patches and n=64 patches). All *in vitro* functional data and quantifications are based on 3 independent experiments with 2-4 replicates for each condition per experiment, except for quantification in Fig 4F and Fig 5F that are based on 2 independent experiments with 3-5 replicates. Western blot data and quantification is based on 3-4 independent experiments (Fig 4A n=4 and Fig 5C,D n=3).
Sample size is indicated in figure legends and/or figures.

- You should report how often each experiment was performed
- You should include a definition of biological versus technical replication
- The data obtained should be provided and sufficient information should be provided to indicate the number of independent biological and/or technical replicates
- If you encountered any outliers, you should describe how these were handled
- Criteria for exclusion/inclusion of data should be clearly stated
- High-throughput sequence data should be uploaded before submission, with a private link for reviewers provided (these are available from both GEO and ArrayExpress)

Please outline where this information can be found within the submission (e.g., sections or figure legends), or explain why this information doesn't apply to your submission:

eLife Sciences Publications, Ltd is a limited liability non-profit non-stock corporation incorporated in the State of Delaware, USA, with company number 5030732, and is registered in the UK with company number FC030576 and branch number BR015634 at the address 1st Floor, 24 Hills Road, Cambridge CB2 1JP | August 2014

For *in vivo* mouse experiments, we state in the figure, the figure legends and/or in the methods section how many biological replicates (number of mice) we analysed from how many independent experiments.

For *in vitro* permeability experiments, we state in the figure legends (Fig 4C, 4F and 5G) how many technical replicates were analysed for 2 or 3 independent experiments.

For *in vitro* image quantifications, we state in the figure legends (Fig 4D, 5B and 5F) how many technical replicates (images) we analysed from 2 or 3 independent experiments.

For *in vitro* Western Blot quantifications, we state in the figure legends (Fig 4A and Fig 5 C,D) how many independent experiments were analysed.

No data were excluded.

Statistical reporting

- Statistical analysis methods should be described and justified
- Raw data should be presented in figures whenever informative to do so (typically when N per group is less than 10)
- For each experiment, you should identify the statistical tests used, exact values of N, definitions of center, methods of multiple test correction, and dispersion and precision measures (e.g., mean, median, SD, SEM, confidence intervals; and, for the major substantive results, a measure of effect size (e.g., Pearson's r, Cohen's d)
- Report exact p-values wherever possible alongside the summary statistics and 95% confidence intervals. These should be reported for all key questions and not only when the p-value is less than 0.05.

Please outline where this information can be found within the submission (e.g., sections or figure legends), or explain why this information doesn't apply to your submission:

Statistical analysis methods are described in each figure legend and summarized in the statistics method section.

We have plotted raw data and single values in all our experiments, except for the percentage of patches (Fig 2-figure supplement 3, 3C, 3E) to simplify the graph. Here, raw data are based on n > 10.

In each figure legend the error bars have been defined (SD or SEM). Exact p-values or adjusted p-values (for multiple comparison) are depicted within the figures.

Additionally, all raw data including the statistical test and the exact p-values are provided as Excel source files.

(For large datasets, or papers with a very large number of statistical tests, you may upload a single table file with tests, Ns, etc., with reference to sections in the manuscript.)

Group allocation

- Indicate how samples were allocated into experimental groups (in the case of clinical studies, please specify allocation to treatment method); if randomization was used, please also state if restricted randomization was applied
- Indicate if masking was used during group allocation, data collection and/or data analysis

Please outline where this information can be found within the submission (e.g., sections or figure legends), or explain why this information doesn't apply to your submission:

Randomization and masking procedures were not used. Allocation of mice into experimental groups was based on genotype. Littermate controls were included. Both female and male mice were included in analyses, and no differences in the phenotype between the genders were observed.

Additional data files ("source data")

- We encourage you to upload relevant additional data files, such as numerical data that are represented as a graph in a figure, or as a summary table
- Where provided, these should be in the most useful format, and they can be uploaded as "Source data" files linked to a main figure or table
- Include model definition files including the full list of parameters used
- Include code used for data analysis (e.g., R, MatLab)

eLife Sciences Publications, Ltd is a limited liability non-profit non-stock corporation incorporated in the State of Delaware, USA, with company number 5030732, and is registered in the UK with company number FC030576 and branch number BR015634 at the address 1st Floor, 24 Hills Road, Cambridge CB2 1JP | August 2014

- Avoid stating that data files are "available upon request"

Please indicate the figures or tables for which source data files have been provided:

Source data files have been provided for Figures 1-5, Fig1_figure supplement 1A, Fig2_figure supplement 1A, 2A,B,3 and Fig3_figure supplement 1B.

eLife Sciences Publications, Ltd is a limited liability non-profit non-stock corporation incorporated in the State of Delaware, USA, with company number 5030732, and is registered in the UK with company number FC030576 and branch number BR015634 at the address 1st Floor, 24 Hills Road, Cambridge CB2 1JP | August 2014
